# Semaphorin 4D Induces Vasculogenic Differentiation of Dental Pulp Stem Cells

**DOI:** 10.3390/dj11070160

**Published:** 2023-06-27

**Authors:** Najla Al Turkestani, Zhaocheng Zhang, Jacques Eduardo Nör

**Affiliations:** 1 Department of Cariology, Restorative Sciences and Endodontics, University of Michigan School of Dentistry, Ann Arbor, MI 48109, USA; alnajla@umich.edu (N.A.T.); zczhang@umich.edu (Z.Z.); 2 Department of Restorative and Aesthetic Dentistry, Faculty of Dentistry, King Abdulaziz University, Jeddah 21589, Saudi Arabia; 3 Department of Biomedical Engineering, University of Michigan College of Engineering, Ann Arbor, MI 48109, USA; 4 Department of Otolaryngology, University of Michigan School of Medicine, Ann Arbor, MI 48109, USA

**Keywords:** regenerative endodontics, angiogenesis, Plexin B1, SEMA4D, dental pulp

## Abstract

This work aimed to evaluate the effect of Semaphorin 4D (SEMA4D) signaling through Plexin B1 on the vasculogenic differentiation of dental pulp stem cells. We assessed the protein expression of SEMA4D and Plexin B1 in dental pulp stem cells (DPSC) from permanent human teeth and stem cells from human exfoliated deciduous (SHED) teeth using Western blots. Their expression in human dental pulp tissues and DPSC-engineered dental pulps was determined using immunofluorescence. We then exposed dental pulp stem cells to recombinant human SEMA4D (rhSEMA4D), evaluated the expression of endothelial cell differentiation markers, and assessed the vasculogenic potential of rhSEMA4D using an in vitro sprouting assay. Lastly, Plexin B1 was silenced to ascertain its role in SEMA4D-mediated vasculogenic differentiation. We found that SEMA4D and Plexin B1 are expressed in DPSC, SHED, and human dental pulp tissues. rhSEMA4D (25–100 ng/mL) induced the expression of endothelial markers, i.e., vascular endothelial growth factor receptor (VEGFR)-2, cluster of differentiation (CD)-31, and tyrosine kinase with immunoglobulin-like and EGF-like domains (Tie)-2, in dental pulp stem cells and promoted capillary-like sprouting in vitro (*p* < 0.05). Furthermore, Plexin B1 silencing abrogated the vasculogenic differentiation of dental pulp stem cells and significantly inhibited capillary sprouting upon exposure to rhSEMA4D. Collectively, these data provide evidence that SEMA4D induces vasculogenic differentiation of dental pulp stem cells through Plexin B1 signaling.

## 1. Introduction

More than five million children and adolescents in the United States experience dental infections and trauma annually, which could lead to pulp necrosis. Deep caries in developing permanent teeth account for approximately 7% of all dental pulp necrosis cases. Furthermore, pulp necrosis subsequently develops in approximately 27% to 80% of children and adolescents exposed to oral trauma [1,2]. Conventional endodontic treatment has been the preferred choice for managing teeth with pulpal and periradicular pathosis for nearly a century [3]. Although this approach preserves natural teeth, it does not fully restore the physiological functions of the dental pulp, such as pulp repair through mineralization, innate immunity, and the sensation of occlusal pressure and pain—factors vital for the long-term survival of teeth. Furthermore, its effectiveness, particularly in relation to the treatment of immature necrotic permanent teeth, has been subject to scrutiny [4,5]. These teeth present distinct complications due to their open apices and thin root walls [6,7,8]. Conventional treatment, i.e., apexification, leads to a loss of vitality in teeth, disruption of root development, and increased susceptibility to root fractures following secondary trauma [2]. Therefore, the pursuit of a biologically driven strategy that supports pulp vitality in immature teeth and promotes continued root development may reinforce the root structure and avert premature tooth loss [3].

The concept of pulp tissue regeneration, which serves as the cornerstone for regenerative endodontics, was originally introduced by Nygaard-Ostby in the 1960s [9]. Subsequently, in 2004, Banchs and Trope proposed a protocol to promote the growth of new soft tissue within the pulp space of infected root canals, after thorough debridement and mechanical creation of blood clot [9,10,11,12]. This technique, particularly when combined with platelet-rich plasma and fibrin, has been shown to enhance the outcomes of immature permanent teeth treatment [13]. Nevertheless, the reliable induction of an appropriate blood clot through over-instrumentation of the canals remains a challenge [14]. Furthermore, achieving consistent radiographic evidence of apical closure or enhancing the strength/length of the root structure has proven variable [1,13]. Notably, the term pulp revascularization has been referenced in the context of regenerative endodontic procedures. However, it should be emphasized that regenerative endodontics requires much more than reestablishing vascularity [9]. Although revascularization is an essential prerequisite for pulp regeneration, a regenerated pulp necessitates odontoblasts, nociceptors, nerve fibers, interstitial fibroblasts, blood vessels, and stem cells to enable replacement of terminally differentiated pulp cells [12,13]. Tissue engineering strategies involving the transplantation of stem cells seeded in scaffolds into the pulp chamber have been an important area of research [7,15].

Preclinical animal studies have demonstrated the capacity of dental stem cells to regenerate the dentin–pulp complex [16,17]. However, a key challenge in dental pulp tissue engineering success is the rapid development of functional local microvascular networks that ensure an adequate supply of nutrients and oxygen to cells involved in the tissue regeneration process and the newly formed tissue [18,19,20]. Within the field of regenerative dentistry, this is challenged by the anatomical restraint of the pulpal space and the fact that all vascularization must access the root canal through the apical foramen network [21]. As a result, the implementation of techniques that enhance vascularization, such as incorporating cells with high angiogenic potential or endothelial progenitor cells into the transplant, is crucial for the success of dental pulp tissue regeneration [22,23]. Leveraging the endothelial differentiation potential of DPSC, it is possible to induce angiogenic characteristics in these cells before their implantation into the root canal. This approach could potentially expedite the process of vascularization during pulp regeneration [24].

Semaphorins are a family of glycoproteins initially identified as axonal guiding molecules during the development of the nervous system [25,26]. Interestingly, membrane-bound SEMA4D was reported to have a potent proangiogenic activity in vitro and in vivo upon binding to Plexin B1 in endothelial cells [27]. Numerous studies have been investigating the role of SEMA4D in promoting cancer angiogenesis and the role of SEMA4D/Plexin B1 signaling in enhancing tumor vascularization [28,29,30,31]. However, their effect on dental pulp stem cells remains unclear. In this study, we evaluated the effect of SEMA4D on the vasculogenic differentiation of dental pulp stem cells. We hypothesized that Plexin B1 regulates SEMA4D-induced vasculogenic differentiation of dental pulp stem cells.

## 2. Materials and Methods

### 2.1. Cell Culture

DPSC (Lonza) and SHED (gift from Songtao Shi) cells were cultured in α-minimum essential medium (Invitrogen, Carlsbad, CA, USA) supplemented with 15% fetal bovine serum (FBS, Invitrogen) and 1% penicillin/streptomycin (Invitrogen) at 37 °C and 5% CO2. When cells reached 80% confluency, they were passed and seeded in 60 mm tissue culture plates. After 24 h, cells were treated with recombinant human SEMA4D (rhSEMA4D; ACRO Biosystems, Newark, DE, USA) at concentrations of 0, 25, 50 and 100 ng/mL for 7 days. Human dermal microvascular endothelial cells (HDMEC; Lonza, Walkersville, MD, USA) were cultured in Endothelial Growth Medium2-MV (EGM2-MV; Lonza) supplemented with 5% FBS (Invitrogen) and 1% penicillin/streptomycin to be used as positive controls. The culture medium was changed every 2–3 days in all experiments.

### 2.2. Western Blot

We collected pulp tissues from extracted non-carious human third molars. We also retrieved DPSC, SHED, and HDMEC from cell culture flasks at 80% confluency. Cells and pulp tissues were lysed in 1% NP-40 buffer. Then, the proteins were electrophoresed in SDS-polyacrylamide gel and transferred to nitrocellulose membranes. The membranes were incubated overnight at 4 °C with anti-human SEMA4D (Bioss Inc., Woburn, MA, USA), anti-human Plexin B1, or anti-human β-actin antibody conjugated with horseradish peroxidase (HRP) (Santa Cruz Biotechnology Inc., Santa Cruz, CA, USA). To verify the endothelial differentiation, we incubated the membranes with the following primary antibodies: rabbit anti-human vascular endothelial growth factor receptor 1 or 2 (VEGFR1, VEGFR2), anti-human Tie2, or a mouse anti-human CD31 (Santa Cruz Biotechnology Inc.). The next day, membranes were incubated with affinity-purified secondary antibodies conjugated with HRP (Jackson Laboratories, West Grove, PA, USA). Lastly, the proteins were visualized using SuperSignal West Pico chemiluminescent substrate (Thermo Fisher Scientific, Rockford, IL, USA).

### 2.3. Tooth Slice/Scaffold Model of Dental Pulp Tissue Engineering

To generate dental pulp-like tissues from DPSCs, we used the tooth slice/scaffold method, as we have described [32]. Briefly, the pulp tissue was carefully removed from 1.3 mm thick tooth slices obtained from extracted non-carious human third molars. The pulp chamber was filled with a porogen (salt) and poly-L-lactic acid (Boehringer Ingelheim, Germany) dissolved in chloroform. Approximately 6 × 10^5^ DPSC cells were suspended in a 1:1 mixture of Matrigel (BD Biosciences, Bedford, MA, USA) and culture medium, and were then seeded in each tooth slice/scaffold that was transplanted into the subcutaneous space of the dorsum of severe combined immunodeficient mice (CB.17 SCID; Charles River, Wilmington, MA, USA). After 3 weeks, tooth slices/scaffolds were retrieved, fixed, demineralized and prepared for histological analyses.

### 2.4. Immunofluorescence

For normal pulp tissue controls, non-carious human third molars extracted for orthodontic reasons were fixed in 10% formalin solution. Alternatively, tooth slices/scaffolds containing the engineered pulp were fixed in 10% buffered formalin and then demineralized. Specimens were dehydrated, embedded in paraffin blocks, and sliced into 5 µm thick sections. The histological sections were deparaffinized and rehydrated. After antigen retrieval, tissue sections were incubated overnight at 4 °C with rabbit anti-human SEMA4D (Bioss Inc.), mouse anti-human Plexin B1 (Santa Cruz Biotechnology Inc.) or non-specific isotype-matched IgG that was used as a negative control. The following day, the sections were incubated with Alexa Fluor 488 goat anti-mouse and goat anti-rabbit IgG secondary antibodies (Thermo Fisher Scientific) and visualized under standard fluorescence microscopy.

### 2.5. In Vitro Sprouting Assay

Approximately 1.5 × 10^4^ DPSC or SHED cells were seeded in each well of a 12-well plate coated with growth-factor reduced Matrigel (BD Biosciences). Cells were cultured in EGM2-MV medium (Lonza) supplemented with 0, 25, 50, and 100 ng/mL of rhSEMA4D (ACRO Biosystems) in triplicates for a total of 7 days. Alternatively, SHED cells stably transduced with shRNA-Scramble or shRNA-Plexin B1 were treated with 0 or 50 ng/mL rhSEMA4D (ACRO Biosystems) for 7 days. To quantify the number of sprouts, 5 random areas/well were marked on the plate. Imaging of those areas was obtained on the 1st, 3rd, 5th, and 7th days of treatment using a microscope (Olympus, Tokyo, Japan), and Image-Pro Plus software. Counting was conducted at 100× magnification.

### 2.6. Plexin B1 Silencing

HEK293T cells were transiently co-transfected with the lentiviral packaging vectors psPAX2, pMD2G (Vector Core, University of Michigan) and shRNA-Plexin B1 or shRNA-Scramble (Santa Cruz Biotechnology Inc.) using the calcium phosphate method. SHED cells were infected with supernatants containing lentivirus and selected with 1 µg/mL of puromycin (InVivogen, San Diego, CA, USA) for at least 1 week. The knockdown of Plexin B1 was verified by Western blot, as described above.

### 2.7. Statistical Analysis

Data for each group were presented as a mean ± standard deviation. Statistical analysis was performed using SPSS version 27.0 (IBM Corp., Armonk, NY, USA). One-way ANOVA combined with Dunnett’s post-hoc test was used to compare the sprouting counts on the 7th day of each treatment group with the control. A *p*-value less than 0.05 was considered statistically significant. Furthermore, a linear regression model was created with the outcome (sprouting counts on the 7th day), predicted variables of concentration and receptor, and the interaction between concentration and receptor.

## 3. Results

### 3.1. Semaphorin 4D and Plexin B1 Are Expressed in Physiological Dental Pulp Tissues and in Tissues Engeneered with DPSCs Seeded in Tooth Slices/Scaffolds

**Figure 1:**
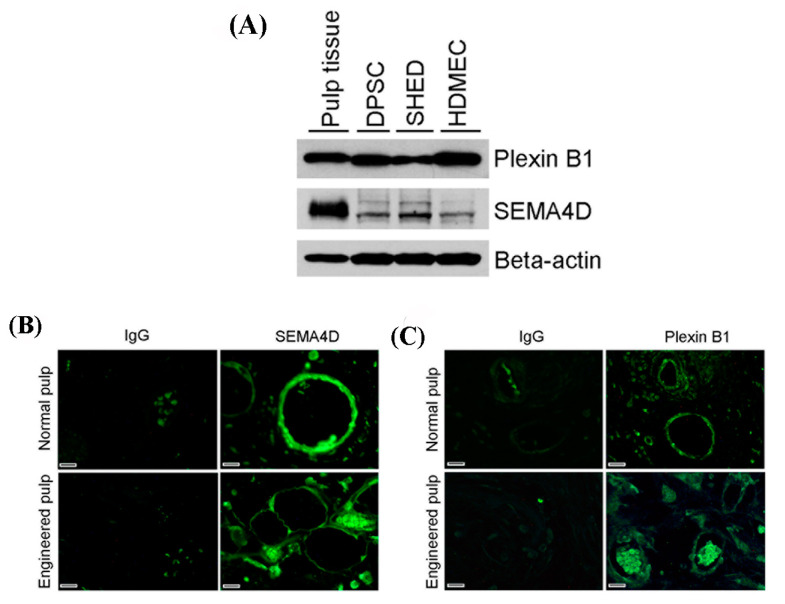
Semaphorin 4D and Plexin B1 expression in the dental pulp. (**A**) Western blot analysis shows the protein expression of SEMA4D and Plexin B1 in lysates from DPSC, SHED cells and human dental pulp tissue. Primary human dermal microvascular endothelial cells (HDMEC) were used as controls. (**B**) Immunofluorescence staining of pulp tissues for SEMA4D or isotype-control IgG. (**C**) Immunofluorescence staining of pulp tissues for Plexin B1 or isotype-control IgG. Top images (**B**,**C**) depict pulp tissues from human non-carious third molars, whereas bottom images show tissues from tooth slice scaffolds seeded with DPSC cells and 3 weeks after transplantation into SCID mice (images taken at 200× magnification, scale bars: 50 μm).

To examine the presence of SEMA4D and Plexin B1 in dental pulp stem cells and pulp tissues, we collected the protein lysates of DPSC, SHED, and pulp tissue from non-carious human third molars. Western blot analyses demonstrated that SEMA4D and Plexin B1 were expressed by DPSC, SHED, and HDMEC (Figure 1A). Interestingly, pulp tissue expressed higher levels of SEMA4D than the cell lines, whereas Plexin B1 expression was comparable. In addition, we performed immunofluorescent staining of tissues obtained from non-carious human third molars and from tooth slice scaffolds seeded with DPSC and retrieved from SCID mice after 3 weeks of implantation. Immunofluorescence showed that SEMA4D and Plexin B1 are expressed in the vascular endothelium of physiological dental pulp tissues and can also be detected in a few other cells (likely fibroblasts) in the engineered dental pulps (Figure 1B,C).

### 3.2. Semaphorin 4D Induces Expresssion of Endothelial Markers in Dental Pulp Stem Cells

**Figure 2:**
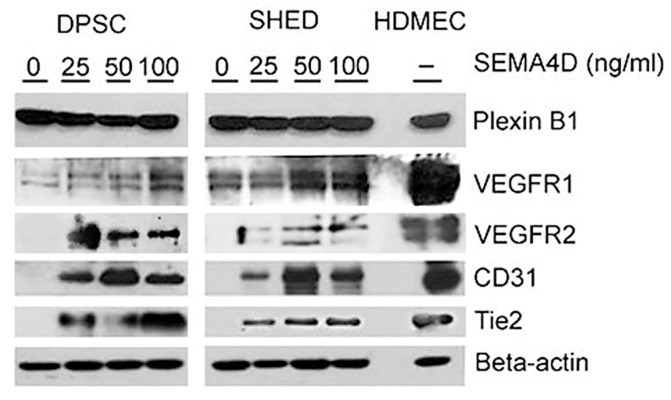
Semaphorin 4D induces expression of endothelial cell markers in dental pulp stem cells. Western blot analysis of DPSC and SHED treated with 0, 25, 50, or 100 ng/mL of rhSEMA4D for 7 days depicts the expression of VEGFR2, CD31, and Tie2. Primary human dermal microvascular endothelial cells (HDMEC) were used as controls.

To initiate an assessment of SEMA4D’s effect on endothelial differentiation of dental pulp stem cells, we subjected these cells to increasing concentrations of rhSEMA4D. After a week, we evaluated the protein expression of several endothelial differentiation markers. Interestingly, rhSEMA4D treatment induced the expression of endothelial markers such as VEGFR2, CD31, and Tie2, along with VEGFR1, which is constitutively expressed in dental pulp stem cells [33]. Each of these proteins was also expressed in HDMECs, which served as our positive controls. As anticipated, the control (untreated) dental pulp stem cells did not express markers of endothelial differentiation. Notably, both DPSC and SHED constitutively express Plexin B1 (Figure 2), which enables a potential mechanism for SEMA4D signaling in undifferentiated cells, a possibility we will explore later in this manuscript.

### 3.3. Semaphorin 4D Enhances Sprouting of Dental Pulp Stem Cells

**Figure 3:**
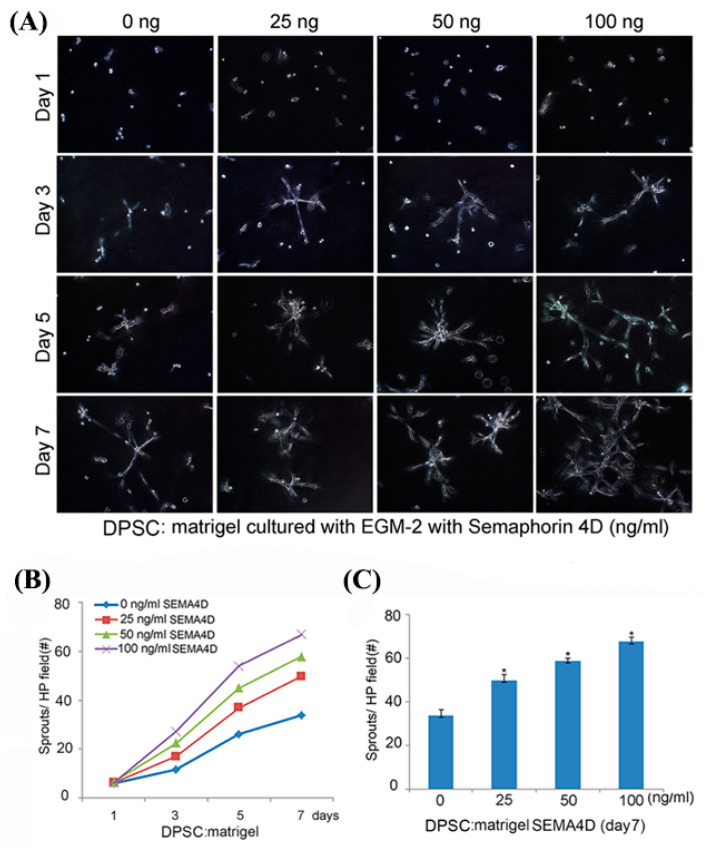
Semaphorin 4D induces DPSC cells to differentiate into sprout-like structures. (**A**) Photomicrographs of DPSC (×100 magnification) seeded in a 12-well plate (1.5 × 10^4^ cells/well) coated with growth factor-reduced Matrigel and cultured with EGM2-MV supplemented with SEMA4D for a week. (**B**) Graph illustrating the average number of sprouts formed by DPSC cultured on 3-D collagen matrices and stimulated with 0–100 ng/mL of rhSEMA4D for 7 days. (**C**) Average number of sprouts developed by DPSC treated with 0–100 ng/mL on the 7th day. Data were analyzed from 15 microscopic fields in triplicate wells per condition and demonstrated as an average ± standard deviation. Values were compared to the control group and statistical significance was determined to be present at *p* < 0.05 (asterisks).

**Figure 4:**
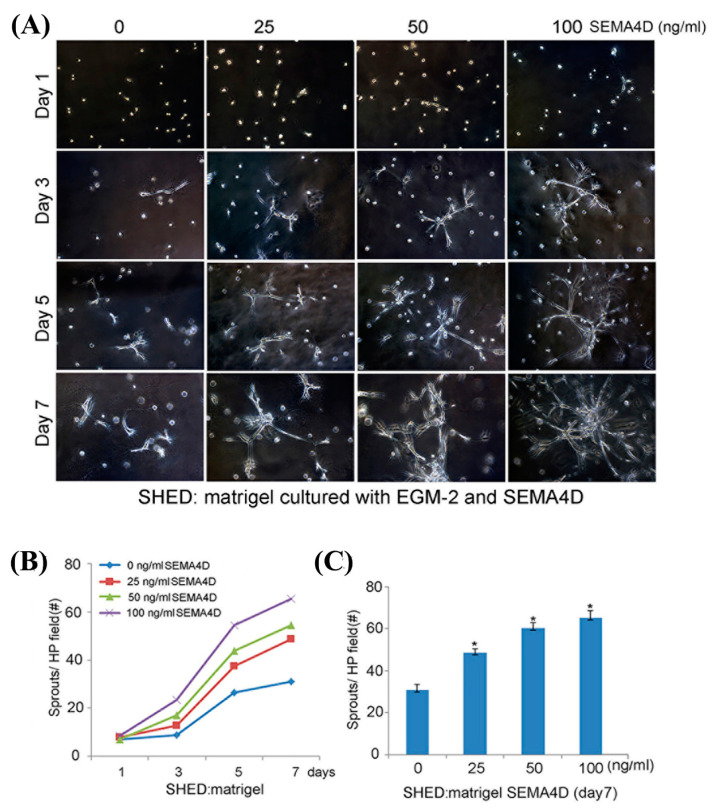
Semaphorin 4D induces SHED cells to differentiate into sprout-like structures. (**A**) Photomicrographs of SHED (×100 magnification) seeded in a 12-well plate (1.5 × 10^4^ cells/well) coated with growth factor-reduced Matrigel and cultured with EGM2-MV supplemented with SEMA4D for a week. (**B**) Graph demonstrating the average number of sprouts formed by SHED cultured on 3-D collagen matrices and stimulated with 0–100 ng/mL of rhSEMA4D for 7 days. (**C**) Average number of sprouts developed by SHED treated with 0–100 ng/mL on the 7th day. Data were analyzed from 15 microscopic fields in triplicate wells per condition and demonstrated as an average ± standard deviation. Values were compared to the control group and the statistical significance was determined at *p* < 0.05 (asterisks).

To further explore the vasculogenic potential of dental pulp stem cells exposed to SEMA4D, we seeded DPSC in 12-well plates coated with growth-factor reduced Matrigel. Cells were cultured in EGM2-MV medium supplemented with 0–100 ng/mL rhSEMA4D. On day 1, we observed that dental pulp stem cells were scattered throughout the gelatinous matrix. By day 3, these cells had started to sprout and form small branches. By day 7, longer branches were noticeable, and vascular networks became apparent. We also observed that sprout number increased in tandem with the escalating dose of rhSEMA4D (Figure 3A). To measure the number of sprouts, we captured images from 5 random areas per well and found that the average sprout count increased with the passage of days and elevation in rhSEMA4D concentration (up to 100 ng/mL). Notably, SEMA4D induced sprout development when compared to control cells cultured in EGM2-MV medium (*p* < 0.05) when we experimented with DPSC (Figure 3B,C). To verify the findings with DPSC from permanent teeth, we replicated these studies with dental pulp stem cells from primary teeth (SHED). We observed that indeed SHED responded very similarly to SEMA4D induction of vasculogenic differentiation, when compared to DPSC cells (Figure 4)

### 3.4. Plexin B1 Signaling Is Required for SEMA4D-Induced Vasculogenic Differentiation of Dental Pulp Stem Cells

**Figure 5:**
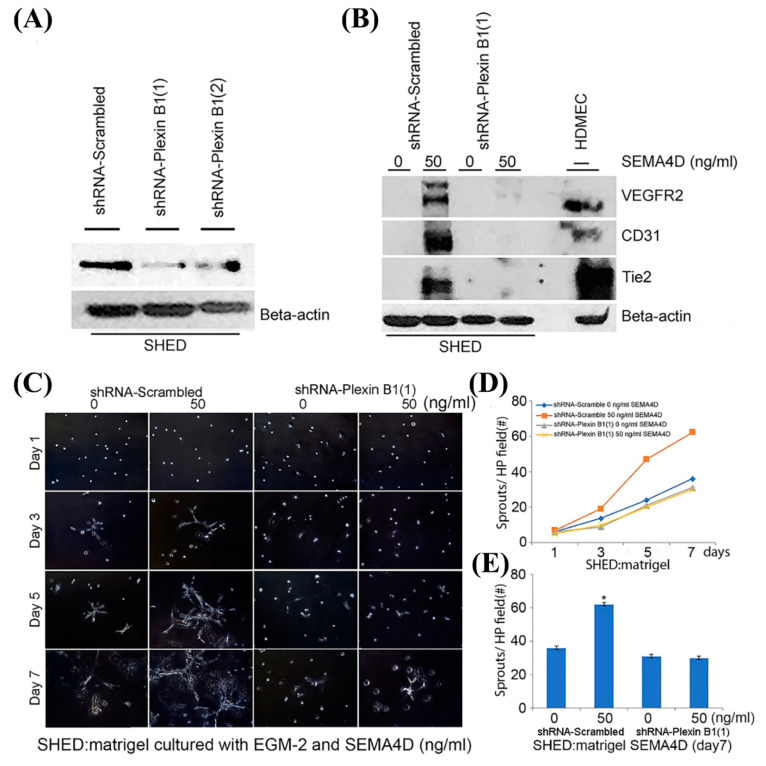
Silencing of Plexin B1 inhibits vasculogenic differentiation of SHED cells. (**A**) SHED stably transduced with shRNA scrambled vector control or two different sequences (1 or 2) of shRNA-PlexinB1. (**B**) Western blot of SHED cells transduced with control shRNA or shRNA-PlexinB1 and treated with 0 or 50 ng/mL of rhSEMA4D for 7 days depicting the expression of the endothelial cell VEGFR2, CD31, and Tie2. However, transduced SHED cells with shRNA-PlexinB1 did not express those markers. (**C**) Photomicrographs of transduced SHED cells with control shRNA or shRNA-PlexinB1 (1) (×100 magnification) seeded in a 12-well plate (1.5 × 10^4^ cells/well) coated with growth-factor reduced Matrigel and cultured with EGM2-MV supplemented with 0 and 50 ng/mL of rhSEMA4D for a week. (**D**) Graph illustrating the average number of sprouts formed by control SHED cells or SHED transduced with shRNA-Plexin B1(1) cultured on Matrigel and treated with 0 or 50 ng/mL of rhSEMA4D for 7 days. (**E**) Average number of sprouts (7th day) generated by control SHED or SHED transduced with shRNA-Plexin B1(1) treated with 0 or 50 ng/mL of rhSEMA4D. Data were analyzed from 15 microscopic fields in triplicate wells per experimental condition and demonstrated as an average ± standard deviation. Values were compared to the control group and the statistical significance was determined at *p* < 0.05 (asterisks).

As we observed that Plexin B1 is constitutively expressed by DPSC and SHED cells (Figure 1A), we hypothesized that this cell membrane receptor plays a role in the SEMA4D-induced vasculogenic differentiation of dental pulp stem cells. To test this hypothesis, we silenced Plexin B1 expression in SHED cells using two sequences of short hairpin-encoding lentiviral vectors. The silencing of Plexin B1 expression was more effective with sequence shRNA-PlexinB1(1) (Figure 5A). Interestingly, Western blots revealed that Plexin B1 silencing abrogated the vasculogenic differentiation of dental pulp stem cells induced by SEMA4D (Figure 5B). Furthermore, we observed a significant decrease in sprouting of Plexin B1-silenced dental pulp stem cells compared to scrambled sequence control cells (Figure 5C–E). SEMA4D induced sprouting of dental pulp stem cells transduced with a control shRNA construct (95% CI = 21.743–30.257; *p* < 0.0005), whereas SEMA4D was unable to induce sprouting in Plexin B1-silenced cells (95% CI = −4.990–3.524; *p* = 0.731). Collectively, these in vitro findings demonstrate that Plexin B1 signaling is integral to SEMA4D-mediated induction of vasculogenic differentiation in dental pulp stem cells.

## 4. Discussion

Regenerative endodontics has led to innovations in the treatment of immature necrotic permanent teeth, as numerous studies have showcased the ability of dental pulp stem cells to regenerate a dentin–pulp-like complex [16,34,35]. However, the challenges posed by the anatomy of the root structure to the vascularization of a pulp-like tissue following stem cell transplantation largely remain unexplored. Here, we demonstrated that SEMA4D/PlexinB1 signaling contributes to the vasculogenic differentiation of dental pulp stem cells. This suggests that activating this pathway could potentially accelerate the vascularization of tissues engineered with these cells.

Semaphorin 4D, a member of the membrane-associated semaphorin family, was the first semaphorin identified in the immune system [36]. SEMA4D expression has been frequently observed in various human cancers as well as craniofacial tissues [37,38,39]. Here, we detected SEMA4D expression in dental pulp stem cells (DPSC), stem cells from human exfoliated deciduous teeth (SHED), and in human dental pulp tissue. This finding may suggest the involvement of SEMA4D in the development and regeneration of dental tissues. Concordantly, Abe and colleagues reported SEMA4D mRNA expression in the dental epithelium and mesenchymal cells of mouse tooth germs at the early bell stage, shifting towards predominant expression in mesenchymal cells at the late bell stage [39]. DPSC and SHED constitute key components of the dental pulp microenvironment and have been recognized for their ability to differentiate into multiple cell types, including those contributing to dental pulp and dentin regeneration [40,41,42]. The expression of SEMA4D within these cells could suggest that this protein may influence their proliferative or migratory abilities [30,43,44]. Interestingly, our fluorescence staining revealed that SEMA4D expression is associated with vascular endothelial cells within dental pulp tissues. Consistent with this observation, SEMA4D expression was identified in endothelial cells of carotid tissue and in HUVEC [45]. Moreover, after a three-week period of implanting DPSC-containing tooth slices/scaffolds into mice, Zhang and colleagues found the majority of DPSC-derived blood vessels were mature and enveloped by pericytes. This may indicate that SEMA4D expression plays a role in the interaction between endothelial and mural cells, a relationship that is vital for vascular development and maturation [46,47].

Plexin B1 is a transmembrane protein that regulates several functions in response to its ligand SEMA4D, including angiogenesis [48]. Previous studies have evaluated Plexin B1′s expression pattern during tooth development in mouse embryos, finding it evident in enamel knots, enamel epithelium, and oral epithelium [49]. Our work reveals Plexin B1 expression in association with endothelial cells in dental pulp tissues. This finding aligns with the previous detection of Plexin B1 in the immunoblot and RNA analyses of porcine aortic endothelial cells [25].

Semaphorin–plexin interactions play a crucial role in key physiological and pathophysiological processes [26]. In particular, these interactions are reported to significantly influence the regulation of blood vessel growth [28,50]. The Gutkind laboratory demonstrated that SEMA4D, cleaved from head and neck squamous cell carcinoma cells (HNSCC), enhances endothelial cell migration by binding to Plexin B1 receptors expressed on these cells [48]. Similarly, Ding and colleagues found that SEMA4D promotes the spreading of human umbilical vein endothelial cells and the formation of tube-like structures, mimicking blood vessels in a Matrigel assay. Additionally, in vivo tumor angiogenic assay also showed that SEMA4D induces an angiogenic response, thereby promoting colorectal cancer growth in a mouse model [51].

Vascular endothelial growth factor (VEGF) is an essential regulator of endothelial cell differentiation, proliferation, migration, and survival. VEGF165 has been shown to prolong the survival of HDMEC in vitro and to enhance the vascularization of severed human dental pulps in vivo [52]. In the current study, we observed that SEMA4D promoted the organization of dental stem cells into sprout-like structures at a rate comparable to that of cells stimulated with VEGF [46]. This finding aligns with previous research, which demonstrated that HUVEC, when cultured with a conditioned medium containing SEMA4D, formed vascular-like structures on Matrigel [53]. Interestingly, Zou and colleagues observed that SEMA4D treatment led to an upregulation of VEGF in DPSC, which enhanced the formation of vascular-like structures by HUVECs [53]. Conversely, the Conrotto group reported no significant difference in expression levels of VEGF-A, angiopoietin-2, and hepatocyte growth factor between unstimulated and SEMA4D-stimulated endothelial cells [54]. These divergent findings highlight the complex nature of SEMA4D’s interactions with other growth factors and their combined effect on vascularization.

Recent studies have highlighted the mechanisms behind SEMA4D signaling and its angiogenic effect in tumor models. They demonstrated that Plexin B1 activation by SEMA4D induces angiogenesis, cytoskeletal reorganization, and the migration of endothelial cells through a RhoA-dependent pathway [31,50,55]. As SEMA4D is highly expressed in HNSCC cells, Basile and colleagues silenced SEMA4D gene expression and found that vascularization of HNSCC tumor xenografts was dramatically decreased [43]. In our study, we found that the vasculogenic differentiation of dental pulp stem cells is halted when SEMA4D signaling is blocked by Plexin B1 gene silencing. Collectively, this work suggests that therapeutic activation of the SEMA4D/PlexinB1 signaling pathway could benefit patients undergoing regenerative endodontics procedures by facilitating rapid establishment of vascular networks following the vasculogenic differentiation of dental pulp stem cells.

## Data Availability

All data related to this study are available within the article.
